# FDM-based 4D printing of sustainable PVA-Cassava fibre composites: a programmable platform for smart gastroretentive drug delivery

**DOI:** 10.1016/j.ijpx.2026.100665

**Published:** 2026-09-13

**Authors:** Pattaraporn Panraksa, Krit Sengtakdaed, Ploynapat Pornngam, Kittisak Jantanasakulwong, Bin Zhang, Claire-Hélène Brachais, Odile Chambin, Pensak Jantrawut

**Affiliations:** a Department of Pharmaceutical Sciences, Faculty of Pharmacy, Chiang Mai University, Chiang Mai 50200, Thailand; b Division of Packaging Technology, School of Agro-Industry, Faculty of Agro-Industry, Chiang Mai University, Chiang Mai 50100, Thailand; c Center of Excellence in Agro Bio-Circular-Green Industry (Agro BCG), Agro-Industry, Chiang Mai University, 155 Mu 2, Mae-hea, Muang, Chiang Mai 50100, Thailand; d Department of Mechanical and Aerospace Engineering, Brunel University London, Uxbridge UB8 3PH, UK; e Laboratoire Interdisciplinaire Carnot de Bourgogne (ICB), UMR 6303 CNRS, Université Bourgogne Europe, 9 avenue Alain Savary, 21078 Dijon, France; f Université Bourgogne Europe, Institut Agro, INRAé UMR PAM joint unit, 1 Esplanade Erasme, 21000 Dijon, France; g Department of Pharmaceutical Technology, Faculty of Health Sciences, Université Bourgogne Europe, 7 bd Jeanne d'Arc, 21079 Dijon, France

**Keywords:** 4D printing, Fused deposition modelling, Gastroretentive drug delivery, Shape-memory polymers, Natural-fibre composites, Programmable drug delivery

## Abstract

With the advancing paradigm of smart and sustainable healthcare, four-dimensional (4D) printing and sustainable polymer composites have attracted increasing interest in the development of advanced drug delivery systems. Incorporating agricultural waste-derived fibres into polymer composites provides a sustainable strategy for producing functional printing materials. In this study, we fabricated 4D-printed gastroretentive platforms by fused deposition modelling (FDM) using poly(vinyl alcohol) (PVA)-Cassava fibre composite filaments. The effects of increasing either Eudragit® NE 30 D or Cassava fibre content (up to 15% *w*/w) on filament flexibility, shape programmability, and drug release were systematically investigated. All developed formulations yielded continuous filaments with smooth extrusion, high structural fidelity, and precise drug loading. While the reference formulation (5% *w*/w fibre, 5% *w*/w Eudragit®; F_5_E_5_) fractured during compression, increasing either component to 15% *w*/w improved flexibility, prevented structural failure, and enabled compression into capsules for convenient oral administration. Following capsule disintegration, the programmed structures recovered their original geometry. Notably, F_15_E_5_ (15% *w*/w fibre, 5% *w*/w Eudragit®) achieved a rapid shape recovery of 93.3 ± 3.6% within 30 min *via* water-triggered polymer relaxation. Both formulations exhibited a pronounced initial release followed by a slower release phase extending to 24 h. Korsmeyer-Peppas analysis indicated anomalous (non-Fickian) transport, consistent with combined diffusion and matrix relaxation/erosion. Overall, this study establishes a 4D printing platform for sequential gastroretentive drug delivery, combining capsule-mediated buoyancy with mechanical expansion, thereby paving the way for the development of next-generation adaptive oral dosage forms with programmable shape transformation and tuneable drug release behaviour.

## Highlights

• A sustainable PVA-Cassava fibre composite filament was developed for 4D printing.
• Increasing Eudragit® or Cassava fibre content to 15% *w*/w overcame matrix brittleness.
• Capsule loading enabled oral dosing and initial buoyancy of 4D-printed constructs.
• Programmed constructs recovered their expanded geometry in simulated gastric fluid.
• 4D-printed constructs with 15% *w*/w Cassava fibre achieved 93.3% shape recovery in 30 min.

## Graphical abstract

**Figure:**
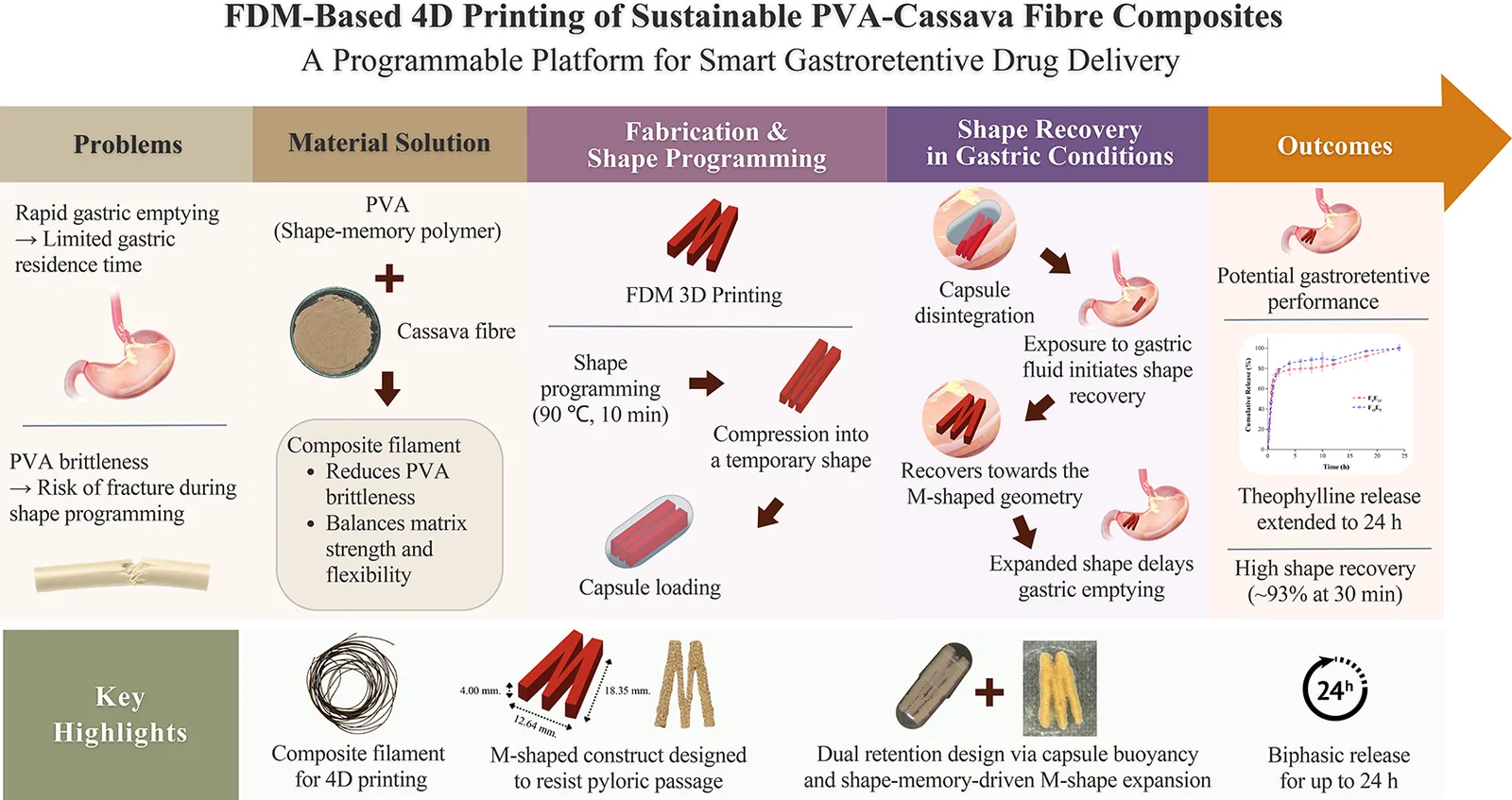

## 1 Introduction

Over the past decade, four-dimensional (4D) printing has emerged as an extension of conventional three-dimensional (3D) printing, integrating digitally designed structures with stimuli-responsive materials (Rastogi and Kandasubramanian, 2019). Unlike conventional 3D printing, which produces static objects, 4D printing enables structures to undergo pre-programmed changes in shape, configuration, or function over time in response to environmental stimuli, such as temperature, pH, light, or moisture (Momeni et al., 2017). The concept has since been widely explored in biomedical applications, including tissue engineering, biomimetic implants, and soft robotics, where shape transformation is used to facilitate minimally invasive implantation, improve conformity to anatomical structures, or provide mechanical support after deployment (Deng et al., 2022; Jin et al., 2018; Mahjoubnia et al., 2024). However, translating this concept into pharmaceutical applications presents a different set of challenges. A 4D-printed dosage form must be made from biocompatible, pharmaceutically acceptable materials and undergo controlled transformation without compromising drug release performance (Yousefi et al., 2026). Moreover, programmable shape transformation should support patient-centred dosage forms that adopt one configuration during administration and another after reaching the target site, thereby addressing the conflicting dimensional requirements of oral administration and therapeutic performance (Panraksa et al., 2025a; Uboldi et al., 2023).

Gastroretentive drug delivery systems (GRDDSs), which are designed to prolong gastric residence time, represent a clear example of this dimensional challenge. Conventional GRDDSs typically rely on floating, swelling, expansion, or mucoadhesion to delay gastric emptying (Mandal et al., 2016; Tripathi et al., 2019). Nevertheless, their performance can be influenced by various physiological conditions in the stomach. Floating systems, for example, require sufficient structural porosity and gastric fluid to maintain buoyancy and their performance can be affected by gastric fluid volume and body position (Ali et al., 2007; Hua, 2020). Similarly, mucoadhesive formulations may undergo premature clearance due to mucus turnover and may also pose a risk of unintended adhesion in the oesophagus (Talukder and Fassihi, 2004). These approaches therefore depend not only on the properties of the dosage form but also on maintaining favourable physiological conditions for buoyancy or sufficient interaction with the gastric mucosa. Among these approaches, expandable systems are particularly attractive because they increase in size or undergo geometric transformation following administration, reducing the likelihood of gastric emptying once their dimensions exceed the pyloric sphincter diameter (∼ 12 mm) (Camilleri et al., 2018). In contrast to floating and mucoadhesive approaches, gastric retention of expandable systems is primarily achieved through a physical size barrier to pyloric passage and may therefore be less sensitive to variations in gastric fluid volume, body position, or mucus turnover that can affect other GRDDS approaches. For example, once an expandable system has reached dimensions larger than the pyloric opening, retention does not require continuous gas generation to maintain a low-density state or persistent polymer-mucus interaction to remain attached to the gastric wall. However, this advantage introduces different design requirements: the system must expand rapidly and reliably after administration, maintain sufficient size and mechanical integrity against gastric contractions during the intended residence period, and subsequently reduce in size or erode to permit safe gastrointestinal transit and minimise the risk of prolonged gastric persistence or bowel obstruction (Bardonnet et al., 2006; Jin et al., 2025). Meeting these design requirements remains challenging for conventional expandable systems, highlighting the potential of 4D printing to enable post-administration shape transformation through design-controlled and programmed material responses rather than passive swelling or mechanical unfolding alone.

Previous studies have established the feasibility of producing compact-to-expanded gastric devices. Flexible and hydrophobic materials such as poly(ε-caprolactone) (PCL) have been used to fabricate complex geometries (*e.g.*, rings or multi-armed stars) that can be tightly folded within capsules and subsequently deployed into an expanded configuration in the gastric environment (Bellinger et al., 2016; Zhang et al., 2015). Although these devices demonstrate a valuable design principle for GRDDS, the slow biodegradation of PCL, while advantageous for residence over days or weeks, may be unsuitable for conventional daily dosing that requires safe elimination within a shorter period. Translation of this concept to 4D-printed GRDDS therefore requires pharmaceutical materials that offer printability, programmable shape change, adequate mechanical integrity, and controlled erosion after drug release.

Poly(vinyl alcohol) (PVA) has become one of the most widely used polymers in FDM-based pharmaceutical printing due to its biocompatibility, thermoplastic processability, hydrophilicity, and dual responsiveness to temperature and water (Mahmoud and Schulz-Siegmund, 2023). These properties allow PVA to be processed into drug-loaded filaments and fabricated into dosage forms with well-defined geometries using FDM (Mallakpour et al., 2022). Its thermo- and water-responsive behaviour also provides a suitable basis for shape programming and recovery under physiologically relevant conditions, making PVA an attractive candidate for 4D-printed pharmaceutical applications. Despite these advantages, the intrinsic properties of PVA remain suboptimal for programmable GRDDS. Its relatively high crystallinity may result in brittle printed constructs, thereby compromising printability and increasing the risk of cracking during shape programming (Palekar et al., 2019; Solórzano-Requejo et al., 2026). In addition, the strong water affinity of PVA promotes rapid hydration, dissolution, and erosion in gastric fluid, which may reduce structural persistence and limit the prolonged gastric residence needed for sustained drug delivery. For example, Melocchi et al. reported that PVA-based expandable dosage forms dissolved rapidly, resulting in complete drug release within 2 h (Melocchi et al., 2019). Therefore, further optimisation of the 4D-printed PVA matrix is required to improve mechanical robustness, structural persistence, and drug release performance for gastroretentive applications.

In this context, the incorporation of natural fibre reinforcements into thermoplastic matrices offers a sustainable and effective strategy for tuning mechanical and physicochemical properties of 4D-printed GRDDS. Cassava pulp, an abundant agricultural by-product from starch processing, represents a renewable lignocellulosic material that can be valorised as a reinforcing filler. The use of such agro-waste-derived materials aligns with circular economy principles by adding value to biomass residues while reducing reliance on fully synthetic excipients. In our previous work, a PVA-Cassava fibre composite filament containing Cassava fibre, Eudragit® NE 30 D, and polyethylene glycol (PEG) 400 was successfully fabricated *via* hot-melt extrusion (HME) and demonstrated suitability for FDM processing (Panraksa et al., 2025b). Building upon this foundation, the present study extends the PVA-Cassava fibre composite into a programmable material platform for FDM-based 4D-printed GRDDS. In the present study, the composite formulation was modified by varying the proportions of Cassava fibre and Eudragit® NE 30 D to investigate their effects on printability, shape memory behaviour, structural integrity, and drug release performance. To further evaluate the feasibility of the 4D-printed GRDDS, theophylline was chosen as the model drug. Its relatively high thermal stability allows it to withstand the elevated temperatures of HME/FDM processing (Tan et al., 2019). In addition, its relatively short half-life, low bioavailability, and rapid absorption occurring primarily in the upper gastrointestinal tract, support its relevance as a model drug for a gastroretentive approach aimed at prolonging gastric residence and drug release (Avbunudiogba et al., 2020; Bhise and Aloorkar, 2008). Within this framework, the 4D-printed constructs were designed to be incorporated into capsules to enable a sequential gastroretentive design, in which capsule-mediated buoyancy is followed by recovery of the predefined permanent geometry after capsule disintegration. To the best of our knowledge, this study is among the first to demonstrate an agricultural fibre-reinforced PVA composite as a programmable FDM-based 4D printing platform for gastroretentive drug delivery, integrating sustainable material design with controlled shape transformation and sequential gastric retention behaviour.

## 2 Materials and methods

### 2.1 Materials and Cassava fibre preparation

Poly(vinyl alcohol) (PVA; Mowiol® 4–88, average molecular weight ∼ 31,000 g/mol, degree of hydrolysis 86.7–88.7 mol%) was purchased from Sigma-Aldrich® (St. Louis, MO, USA). Polyethylene glycol (PEG 400), used as a plasticiser, was obtained from Union Science Co., Ltd. (Chiang Mai, Thailand). Theophylline anhydrous (C_7_H_8_N_4_O_2_, purity ≥99%), serving as the model drug, was purchased from Acros Organics (Geel, Belgium). Eudragit® NE 30 D was obtained from Röhm GmbH & Co. KG (Darmstadt, Germany) and incorporated as a water-insoluble matrix modifier to improve matrix cohesion and structural persistence. Calcium stearate, used as a lubricant, was purchased from MySkinRecipes (Bangkok, Thailand). Size 0 hard hydroxypropyl methylcellulose (HPMC) capsules were obtained from Capsule Products Co., Ltd. (Bangkok, Thailand) and used to contain the programmed temporary constructs. Hydrochloric acid (1 N) was purchased from RCI Labscan Limited (Bangkok, Thailand).

Cassava fibre was prepared from Cassava pulp waste following a previously reported method (Panraksa et al., 2025b). Briefly, the pulp underwent starch removal and alkaline treatment to isolate short lignocellulosic fibres. The fibres were subsequently washed, dried at 70 °C, passed through a No. 325 sieve (nominal aperture approximately 45 μm), and stored in a desiccator until use.

### 2.2 Composite formulation design and hot-melt extrusion of filaments

#### 2.2.1 Formulation design

Three theophylline-loaded PVA-Cassava fibre composite formulations were prepared to investigate the influence of composition on filament processability, FDM printability, shape transformation, and *in vitro* gastroretentive performance. The P_10_F_5_E_5_T_5_ formulation, previously identified as an optimal composition in our earlier study (Panraksa et al., 2025b), was included as the reference formulation. The code P_10_F_5_E_5_T_5_ represents 10% *w*/w PEG 400, 5% *w*/w Cassava fibre, 5% *w*/w Eudragit® NE 30 D, and 5% *w*/w theophylline. In the present study, this formulation is referred to as F_5_E_5_ for simplicity. Two modified formulations were developed by increasing either the Eudragit® NE 30 D content or Cassava fibre content to 15% *w*/w, while maintaining constant concentrations of PEG 400, calcium stearate, and theophylline. The compositions of all three formulations are summarised in Table 1.

**Table 1:** Composition of theophylline-loaded PVA-Cassava fibre composite formulations (% w/w).

Formulation code	PVA	PEG400	Cassava fibre	Eudragit® NE 30 D	Calcium stearate	Theophylline anhydrous
F_5_E_5_	74.0	10.0	5.0	5.0	1.0	5.0
F_5_E_15_	64.0	10.0	5.0	15.0	1.0	5.0
F_15_E_5_	64.0	10.0	15.0	5.0	1.0	5.0

Note: Eudragit® NE 30 D content refers to the amount of the commercial aqueous dispersion used in the formulation.

#### 2.2.2 Preparation of composite filaments

Composite filaments were prepared by HME from PVA-Cassava fibre blends formulated according to Table 1. The solid components were first premixed by geometric dilution using a mortar and pestle to ensure uniform distribution of the drug and fibre within the PVA matrix. PEG 400 and Eudragit® NE 30 D were then gradually incorporated, and mixing was continued for 10 min to obtain homogeneous composite blends. Prior to extrusion, the blends were dried at 50 °C overnight. In this study, the dried blends were extruded using a custom-built single-screw extruder fitted with a 1.75 mm die developed by the Biomedical Engineering Institute, Chiang Mai University, as described in our previous work (Panraksa et al., 2025b). Extrusion parameters were selected for each formulation based on preliminary trials to achieve continuous extrusion and obtain filaments with a suitable and consistent diameter for subsequent FDM printing. The screw speed and conveyor belt speed were adjusted accordingly to accommodate differences in extrusion behaviour among the formulations, while the pre-heating and extrusion temperatures were maintained at 195 and 200 °C, respectively (Table 2).

**Table 2:** Hot-melt extrusion parameters for PVA-Cassava fibre composite filaments.

Formulation code	Pre-heating temperature (°C)	Extrusion temperature (°C)	Screw speed (rpm)	Conveyor belt speed (mm/s)
F_5_E_5_	195	200	7.5	2.17
F_5_E_15_	195	200	12	3.33
F_15_E_5_	195	200	12	3.33

### 2.3 Characterisation of composite filaments

#### 2.3.1 Filament diameter, appearance, and morphology

Filament diameter was measured using a digital calliper (BEC, United Hardware Co., Ltd., Bangkok, Thailand) at 10-cm intervals along the extruded filament length. At least 10 measurements were recorded for each formulation.

Filament surface morphology was initially examined using a portable USB digital microscope (Yao, Shenzhen, China) equipped with an HD CMOS sensor and eight integrated LED lights. The microscope was positioned at a fixed distance of 3.0 cm from the filament surface, and images were captured using camera software for Windows 10 (version 2025.2505.2.0, Microsoft Corporation, Redmond, WA, USA), at a resolution of 640 pixels. The microstructure of the composite filaments was further analysed using a benchtop scanning electron microscope (SEM; JCM-7000 NeoScope™, JEOL Ltd., Tokyo, Japan). Samples were mounted on aluminium stubs using conductive carbon tape (Nisshin EM Co., Ltd., Tokyo, Japan) and sputter-coated with gold using an automated coater (JEOL Smart Coater, JEOL Ltd., Tokyo, Japan). SEM imaging was performed under high-vacuum mode using a secondary electron detector at an accelerating voltage of 15.0 kV. Surface and cross-sectional images were collected at magnifications of 100× and 50×, respectively, to evaluate surface texture, internal morphology, and structural irregularities, such as voids and particulate aggregates.

#### 2.3.2 Fourier transform infrared spectroscopy

Fourier transform infrared (FTIR) spectroscopy was conducted to characterise the chemical properties of the raw materials (PVA, Cassava fibre, Eudragit® NE 30 D, and theophylline) and composite filaments and to evaluate potential chemical changes induced by HME. Samples were analysed using an FTIR spectrometer (INVENIO®, Bruker Corporation, Billerica, MA, USA) equipped with an attenuated total reflectance (ATR) accessory. Spectra were collected over the wavenumber range of 4000–400 cm^−1^ at a resolution of 4 cm^−1^ using 32 scans per sample.

#### 2.3.3 Mechanical properties

The mechanical properties of the extruded composite filaments were evaluated using a texture analyser (Stable Micro Systems, Surrey, UK) equipped with a 50 kg load cell. Filament samples (∼50 mm in length) were secured between tensile grips and tested at 25 °C with a crosshead speed of 2 mm/min. A displacement of 10 mm was set for consistent comparison between the different filament formulations. Force-displacement data were recorded using TEConnect software version 8.0. At least three samples were tested for each formulation.

Prior to tensile testing, the diameter of each filament sample was measured at five positions using a digital calliper, and the mean diameter was used to calculate its initial cross-sectional area. The recorded force-displacement data were converted to stress-strain curves based on the calculated cross-sectional area and sample length.

Maximum force ($F_{\max}$) was defined as the highest force recorded during the tensile test. Tensile strength (TS) was calculated as the maximum stress of each individual filament sample according to Eq. (1):

(1) $$\mathrm{TS}=\sigma_{\max}=\frac{F_{\max}}{A}$$

where $F_{\max}$ is the maximum force recorded during the tensile test and A is the initial cross-sectional area of the filament sample.

Apparent Young's modulus ($E_{\mathrm{app}}$) was determined from the slope of the stress-strain curve within the predefined linear elastic region according to Eq. (2):

(2) $$E_{\mathrm{app}}=\frac{∆\sigma }{∆\varepsilon }$$

where ∆σ and ∆ε represent the change in stress and strain, respectively, within the selected linear region.

#### 2.3.4 Theophylline content and distribution uniformity in filaments

Theophylline content and distribution uniformity in the composite filaments were evaluated to confirm drug loading accuracy and homogeneity after HME. Filament were sampled from the beginning, middle and end sections of each batch. Each sample (∼100 ± 5 mg) was dissolved in distilled water under magnetic stirring for 2 h and filtered through a 0.45 μm membrane filter (Labfil®, Zhejiang ALWSCI Technologies Co., Ltd., Zhejiang PR, China). The filtrate was appropriately diluted, and theophylline concentration was quantified by UV–Vis spectrophotometry (UV-2600i, Shimadzu Corporation, Kyoto, Japan) at 271 nm using a calibration curve prepared in distilled water (y = 0.0551x − 0.0974, R^2^ = 0.9986, where x is the theophylline concentration (μg/mL) and y is absorbance). The measured theophylline content was expressed as a percentage of the theoretical drug content of each formulation. At least three samples were analysed per formulation.

### 2.4 Design, FDM printing and structural characterisation of 4D-printed constructs

#### 2.4.1 Design of permanent geometry

**Fig. 1:**
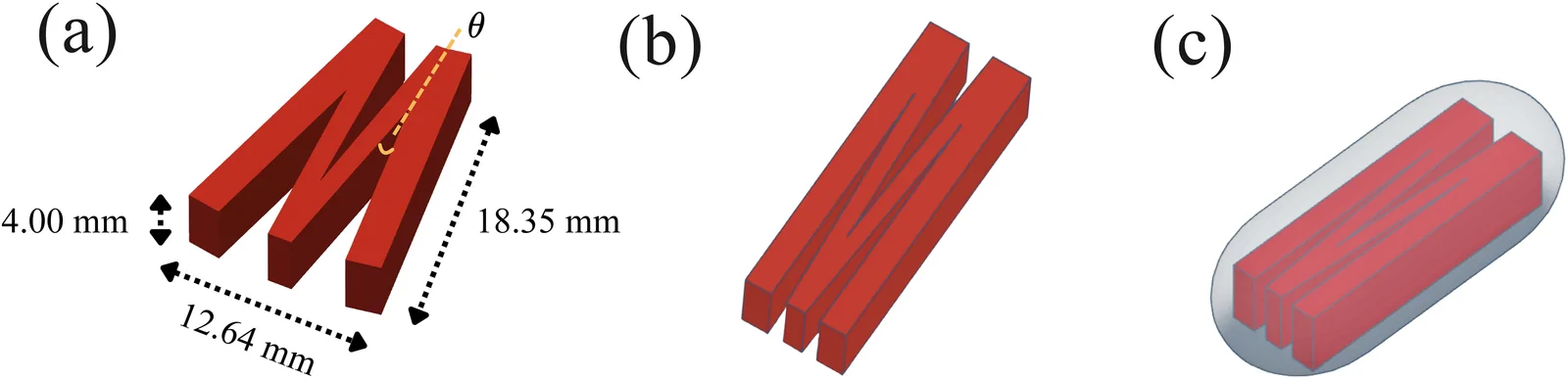
Design and shape programming concept of 4D-printed GRDDS: (a) CAD model of the permanent geometry, (b) compact temporary configuration, and (c) programmed construct loaded into a capsule.

The permanent geometry of the 4D-printed construct was designed as an expanded M-shaped structure using computer-aided design (CAD) software (Tinkercad® 2024, Autodesk Inc., San Francisco, CA, USA), with dimensions of approximately 18.35 mm in length, 12.64 mm in width, and 4.00 mm in thickness (Fig. 1a). These dimensions refer to the unfolded configuration before thermomechanical programming into the compact temporary shape (Fig. 1b) for capsule loading (Fig. 1c). Following exposure to simulated gastric conditions, the construct was intended to recover towards its permanent, expanded geometry. The dimensions of the permanent geometry were selected with consideration of the reported pyloric sphincter diameter (approximately 12 ± 7 mm) (Mahmoud and Schulz-Siegmund, 2023), with the aim of supporting gastric retention following shape recovery.

#### 2.4.2 FDM printing of 4D-printed constructs

The predesigned CAD model was exported as a stereolithography (.STL) file and imported into PrusaSlicer software version 2.9.2 (Prusa Research, Prague, Czech Republic) to generate the FDM printing path. The 4D-printed constructs were fabricated using an Original Prusa Mini+ printer equipped with a 0.4 mm nozzle (Prusa Research, Prague, Czech Republic). Printing parameters were set as follows: nozzle temperature of 200 °C, bed temperature of 60 °C, layer height of 0.2 mm, printing speed of 10 mm/s, rectilinear infill pattern, and 100% infill density. The measured mean filament diameter of each formulation was also specified in slicing software to optimise material extrusion. Printability was qualitatively assessed based on filament feeding continuity, layer deposition, and the presence of visible printing defects, including warping, incomplete deposition, and structural collapse. Following printing, the 4D-printed constructs were stored in vacuum-sealed bags at room temperature (25 ± 2 °C) until further characterisation and testing.

#### 2.4.3 Dimensional accuracy and drug content of 4D-printed constructs

The dimensional accuracy of the 4D-printed constructs was evaluated by measuring their length, width and thickness using a digital calliper. At least five constructs per formulation were analysed. Dimensional accuracy was calculated as the percentage ratio between the measured dimensions and corresponding CAD dimensions, with values approaching 100% indicating higher printing fidelity.

Theophylline content was determined in at least three 4D-printed constructs per formulation using the method described in Section 2.3.4. Prior to analysis, the mass of each construct was recorded using an analytical balance (LAB 124i, Adam Equipment Co. Ltd., Milton Keynes, UK). Drug content was expressed as a percentage of the theoretical drug content to evaluate dose accuracy following FDM processing.

#### 2.4.4 Solid-state and thermal characterisation of 4D-printed constructs

X-ray diffraction (XRD) analysis was performed to evaluate changes in the crystalline state of the polymer matrix and theophylline following HME-FDM processing. Diffraction patterns of raw PVA, raw theophylline, and 4D-printed constructs were recorded using an X-ray diffractometer (PANalytical Empyrean, Malvern Panalytical Ltd., Worcestershire, UK) with Cu Kα radiation operated at 45 kV and 30 mA. Measurements were performed over a 2θ range of 5–60° using a step size of 0.013° in continuous-scan mode.

Differential scanning calorimetry (DSC) was performed using a differential scanning calorimeter (Discovery DSC, TA Instruments, New Castle, DE, USA) to evaluate thermal transitions of 4D-printed constructs. Approximately 10 mg of each sample was sealed in Tzero aluminium pans (TA Instruments, New Castle, DE, USA) and heated from 10 to 210 °C at a heating rate of 20 °C/min under a nitrogen purge (200 mL/min). The thermograms were analysed to determine the melting behaviour, the crystallinity of the PVA phase, melting peak temperature ($T_{m}$) and apparent melting enthalpy ($∆H_{m}$) of the 4D-printed constructs after HME-FDM processing.

### 2.5 Shape programming, capsule loading and *in vitro* shape-recovery behaviour

#### 2.5.1 Shape programming and capsule loading

The 4D-printed constructs were programmed into temporary configurations by thermomechanical treatment. Briefly, constructs were heated in a hot-air oven (Memmert GmbH + Co. KG, Büchenbach, Germany) at 90 °C for 10 min and compressed into a capsule-compatible configuration using a 100 g calibration weight. The deformed constructs were then cooled at 4 ± 0.5 °C to fix the temporary shape. Subsequently, the programmed constructs were loaded into size 0 HPMC capsules and stored in airtight containers until further evaluation.

#### 2.5.2 Shape recovery, expansion and *in vitro* floating observation

Capsule-loaded 4D-printed constructs were immersed in approximately 50 mL of 0.1 N HCl at 37 ± 0.5 °C to evaluate their *in vitro* transformation and gastroretentive behaviour. Capsule disintegration, floating behaviour, and construct expansion were visually monitored at predetermined time intervals (5, 10, 15, 20, and 30 min) and digital images were captured to document the transformation process.

Shape recovery was quantified based on the angles formed at the two *V*-shaped outer bends of the M-shaped construct, with a representative angular measurement position illustrated in Fig. 1a. For each construct, both outer-bend angles were measured in the permanent, temporary, and recovered states, and the mean of the two angles was used for shape-recovery analysis. Three constructs were analysed for each formulation. The permanent angle ($\theta_{p}$) was measured before programming, the temporary angle ($\theta_{t}$) after deformation and shape fixation, and the recovered angle ($\theta_{r}$) after exposure to simulated gastric conditions for the specified recovery time.

Digital images were captured from a fixed camera position at a distance of approximately 10 cm above the liquid surface, with the camera oriented perpendicular to the sample plane. The same imaging conditions and angular reference positions were maintained throughout the measurements to minimise variability associated with image projection. The angles were measured from digital images using ImageJ software (version 1.54 g, National Institutes of Health, Rockville, MD, USA), and the shape recovery ratio was calculated according to Eq. (3):

(3) $$\mathrm{Shape recovery}\;\left(\%\right)=\frac{\left|\theta_{r}-\theta_{t}\right|}{\left|\theta_{p}-\theta_{t}\right|}\times 100$$

### 2.6 Swelling behaviour of 4D-printed constructs

The swelling behaviour of the 4D-printed constructs was evaluated using an image-based method adapted from a previously reported study (Zhang et al., 2021). The 4D-printed constructs were immersed in approximately 50 mL of 0.1 N HCl maintained at 37 ± 0.5 °C and digital images were captured at predetermined time intervals under fixed imaging conditions. The projected surface area of each construct, including the visible gel layer, was quantified using ImageJ software. The apparent swelling ratio was calculated according to Eq. (4):

(4) $$\mathrm{Apparent swelling ratio}\;\left(\%\right)=\left(\frac{A_{t}-A_{0}}{A_{0}}\right)\times 100$$

where $A_{0}$ is the initial projected surface area before immersion, $A_{t}$ is the projected surface area measured at time t, and t is the immersion time.

### 2.7 *In vitro* theophylline release and release kinetic analysis

The *in vitro* release of theophylline from capsule-loaded 4D-printed constructs was evaluated in 500 mL of 0.1 N HCl maintained at 37 ± 0.5 °C using a USP Apparatus II dissolution tester (Hanson SR8-Plus™, Hanson Research Corporation, Chatsworth, CA, USA) operated at 50 rpm. The dissolution-medium volume was selected based on previously reported *in vitro* dissolution conditions for gastroretentive drug delivery systems (Gupta et al., 2025). Each formulation was tested in triplicate.

At predetermined time intervals (2.5, 5, 10, 15, 20, 30, 40, 50 min and 1, 1.5, 2, 4, 6, 8, 10, 12, 18, 24 h), 5 mL aliquots were withdrawn and filtered through a 0.45 μm nylon syringe filter. The withdrawn volume was immediately replaced with an equal volume of pre-warmed 0.1 N HCl to maintain a constant dissolution medium volume. Subsequently, the filtrates were appropriately diluted and analysed by UV–Vis spectrophotometry at 270 nm. Theophylline concentration was determined using a calibration curve prepared in 0.1 N HCl (y = 0.0551x − 0.0974, R^2^ = 0.9995, where x and y represent the theophylline concentration in μg/mL and absorbance, respectively). Cumulative drug release was corrected for sampling and medium replacement. The cumulative percentage of theophylline released at each time point was calculated relative to the theoretical drug content of each construct and plotted against time.

The drug release data were further analysed using the Korsmeyer–Peppas model to investigate the release mechanism of the 4D-printed constructs. The model was expressed according to Eq. (5):

(5) $$\frac{M_{t}}{M_{\infty }}=k_{p}\times t^{n}$$

where $\frac{M_{t}}{M_{\infty }}$ is the fraction of drug released at time t, $k_{p}$ is the kinetic constant incorporating structural and geometric characteristics of the dosage form, and n is the release exponent.

### 2.8 Statistical analysis

All experiments were performed at least in triplicate unless otherwise stated. Data were expressed as mean ± standard deviation (SD). Statistical analyses were performed using SPSS Statistics software 17.0 (SPSS Inc., Chicago, IL, USA). Differences among formulations were evaluated using one-way analysis of variance (ANOVA) followed by an appropriate *post hoc* test. A *p*-value of less than 0.05 was considered statistically significant.

## 3 Results and discussion

### 3.1 Filament characteristics and FDM processability

#### 3.1.1 Physical characteristics and morphology

All formulations were successfully processed into continuous filaments under their respective optimised hot-melt extrusion conditions. The mean filament diameters of F_5_E_5_, F_5_E_15_ and F_15_E_5_ were found to be 1.57 ± 0.03, 1.54 ± 0.04 and 1.52 ± 0.02 mm, respectively. Statistical analysis showed that the high-fibre formulation (F_15_E_5_) had a significantly smaller mean filament diameter than the reference formulation F_5_E_5_ (*p* < 0.05), whereas no significant difference was observed between F_15_E_5_ and F_5_E_15_.

Despite optimisation of the extrusion parameters, the final filament diameters of all formulations remained below the nominal die diameter (1.75 mm). This may be attributed to the post-die behaviour of the composite melts. After exiting the die, the extrudate may undergo limited die swell, together with axial elongation during take-up and dimensional contraction during cooling, resulting in a smaller final filament diameter (Aho et al., 2019; Bandari et al., 2021). Furthermore, the smaller diameter observed for F_15_E_5_ may be related to the higher fibre loading, as the presence of fibres can restrict polymer chain relaxation after extrusion, thereby reducing the outward expansion of the extruded strand after leaving the die (Tekinalp et al., 2014). Nevertheless, the obtained filament diameters were consistent within each formulation, as indicated by the low SD values. Although the diameters were smaller than the nominal die size, this difference did not compromise FDM processing. The experimentally measured mean filament diameter of each formulation was entered into PrusaSlicer software as the filament diameter setting so that material extrusion could be adjusted according to the actual filament dimensions. Using these settings, all formulations were successfully printed without feeding interruptions, demonstrating sufficient dimensional consistency and processability for FDM fabrication.

**Fig. 2:**
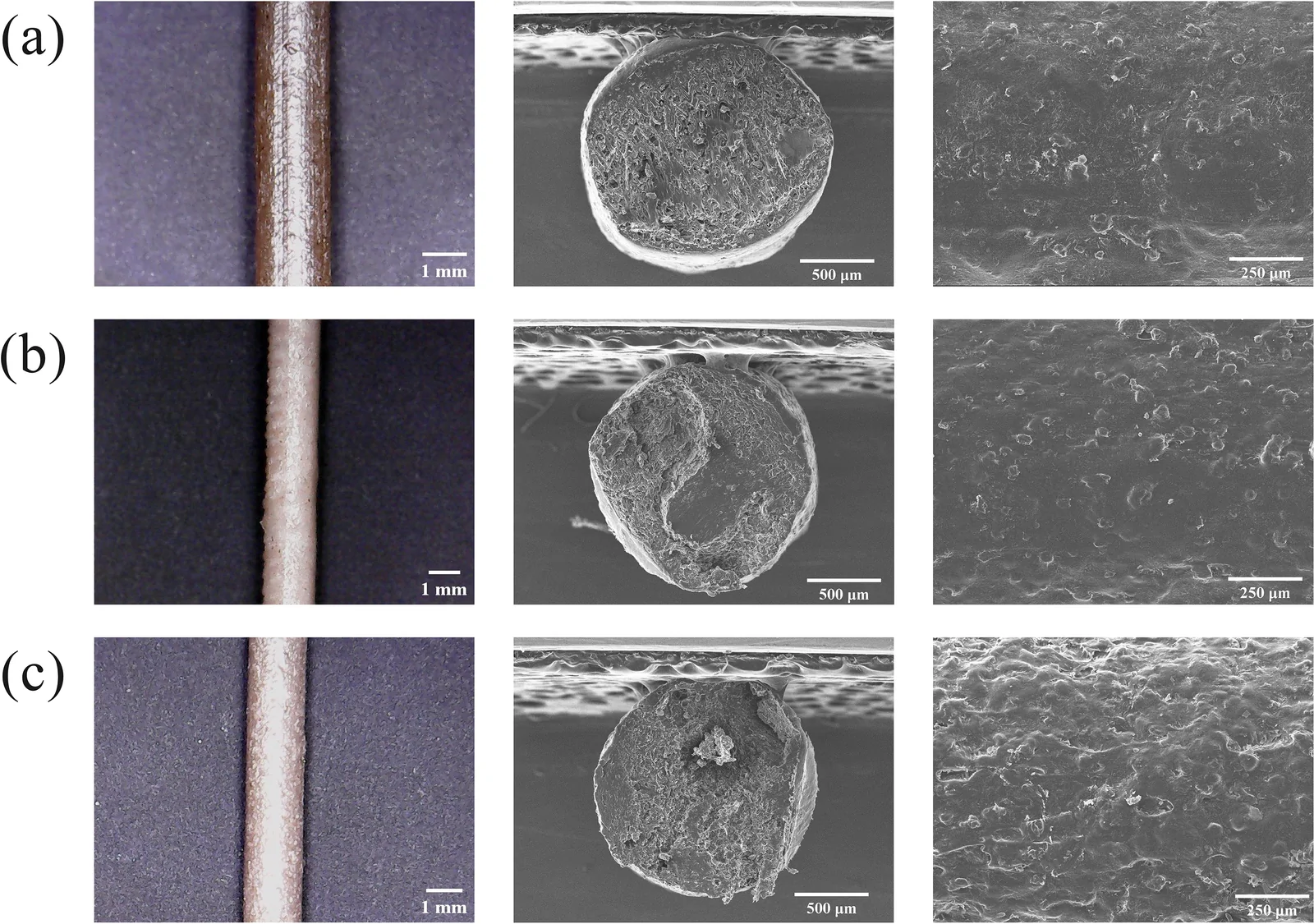
Digital microscope images (left), cross-sectional (middle), and surface (right) SEM micrographs of (a) F_5_E_5_, (b) F_5_E_15_, and (c) F_15_E_5_ composite filaments.

For the morphological evaluation, all extruded filaments exhibited a brown to dark brown appearance under digital microscopy (Fig. 2). The filaments showed uniform cylindrical shapes without visible cracks, large air bubbles, or discontinuities. Microstructural evaluation *via* SEM (Fig. 2) further confirmed the absence of major surface defects and cross-sectional irregularities, suggesting that the selected extrusion conditions were appropriate for producing continuous composite filaments with acceptable structural integrity.

Compared to the reference formulation (F_5_E_5_), increasing the Eudragit® NE 30 D content to 15% *w*/w (F_5_E_15_) did not result in an obvious change in surface morphology or cross-sectional appearance. In contrast, increasing the Cassava fibre content to 15% *w*/w (F_15_E_5_) resulted in a visibly rougher and more textured filament surface. This change was likely associated with greater fibre exposure at the filament surface and/or increased heterogeneity within the composite matrix. A similar increase in surface roughness has also been previously reported for polymer composites containing higher contents of agricultural waste-derived fibres, including polycaprolactone (PCL)/cocoa shell waste composites (Tran et al., 2017) and thermoplastic copolyester (TPC)/soybean hull fibre composites (Balla et al., 2021). Nevertheless, despite the more irregular surface appearance, F_15_E_5_ remained continuous and could be processed *via* FDM without feeding interruptions. This indicates that the increased surface roughness associated with higher fibre loading was insufficient to negatively affect filament handling and printing performance, as the filament remained dimensionally consistent and sufficiently flexible to pass through the feeding gears and nozzle pathway.

#### 3.1.2 FTIR characteristics, tensile performance, and drug content uniformity of the composite filaments

**Fig. 3:**
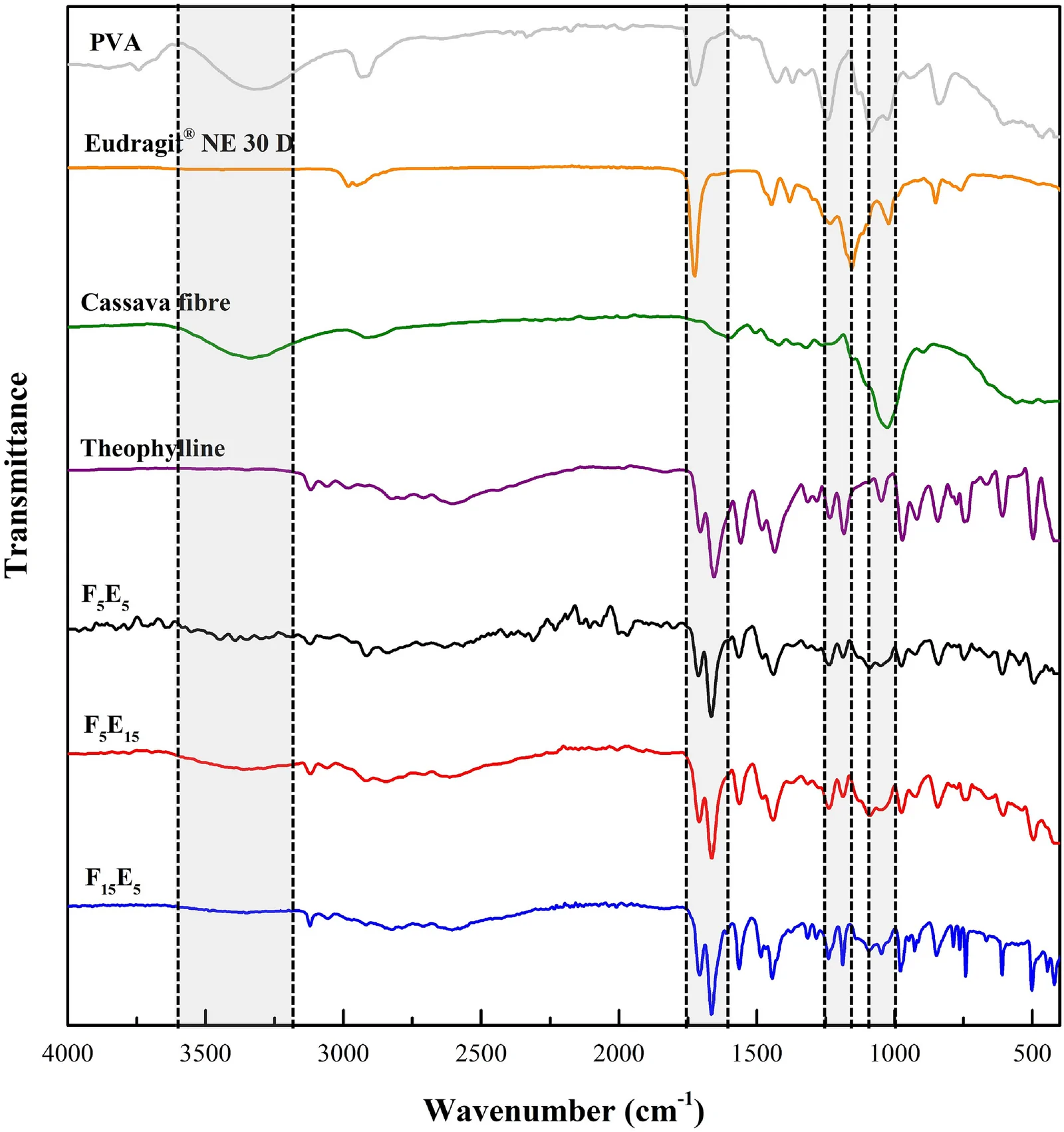
FTIR spectra of raw materials and PVA-Cassava fibre composite filaments.

FTIR analysis was performed to compare the chemical characteristics of the raw materials and extruded composite filaments and to evaluate potential changes following hot-melt extrusion. As shown in Fig. 3, pure PVA exhibited a broad absorption band at 3500–3200 cm^−1^, corresponding to O—H stretching, together with a C—H stretching band at approximately 2935 cm^−1^. A distinct absorption peak near 1724 cm^−1^ was also attributed to the C=O stretching of residual acetate groups in PVA (Mansur et al., 2008; Vilamová et al., 2019). Eudragit® NE 30 D exhibited a strong ester carbonyl band at approximately 1726 cm^−1^ and characteristic C—O stretching bands within the 1250–1150 cm^−1^ region (Lin et al., 2001). Cassava fibre showed a broad O—H stretching band at approximately 3340 cm^−1^ and a C—O stretching band near 1025 cm^−1^, which are characteristic of cellulose-rich materials (Huang et al., 2018; Okrathok et al., 2022). Theophylline showed a characteristic N—H stretching absorption at approximately 3120 cm^−1^ and carbonyl absorption bands at 1654 and 1705 cm^−1^ (Madarász et al., 2002).

Following hot-melt extrusion, the spectra of F_5_E_5_, F_5_E_15_, and F_15_E_5_ retained the characteristic absorption regions of the individual components, including the broad O—H stretching region (3600–3200 cm^−1^), overlapping carbonyl region (1750–1640 cm^−1^), and the fingerprint within the 1250–1000 cm^−1^ region. Changes in band shape and intensity were observed, particularly in the broad O—H stretching and fingerprint regions, which may reflect changes in the local molecular environment and intermolecular interactions within the composite matrix following blending and thermal processing. The relatively prominent theophylline-related absorptions may also overlap with signals from the other formulation components in several spectral regions. No prominent new absorption bands were observed after extrusion.

**Fig. 4:**
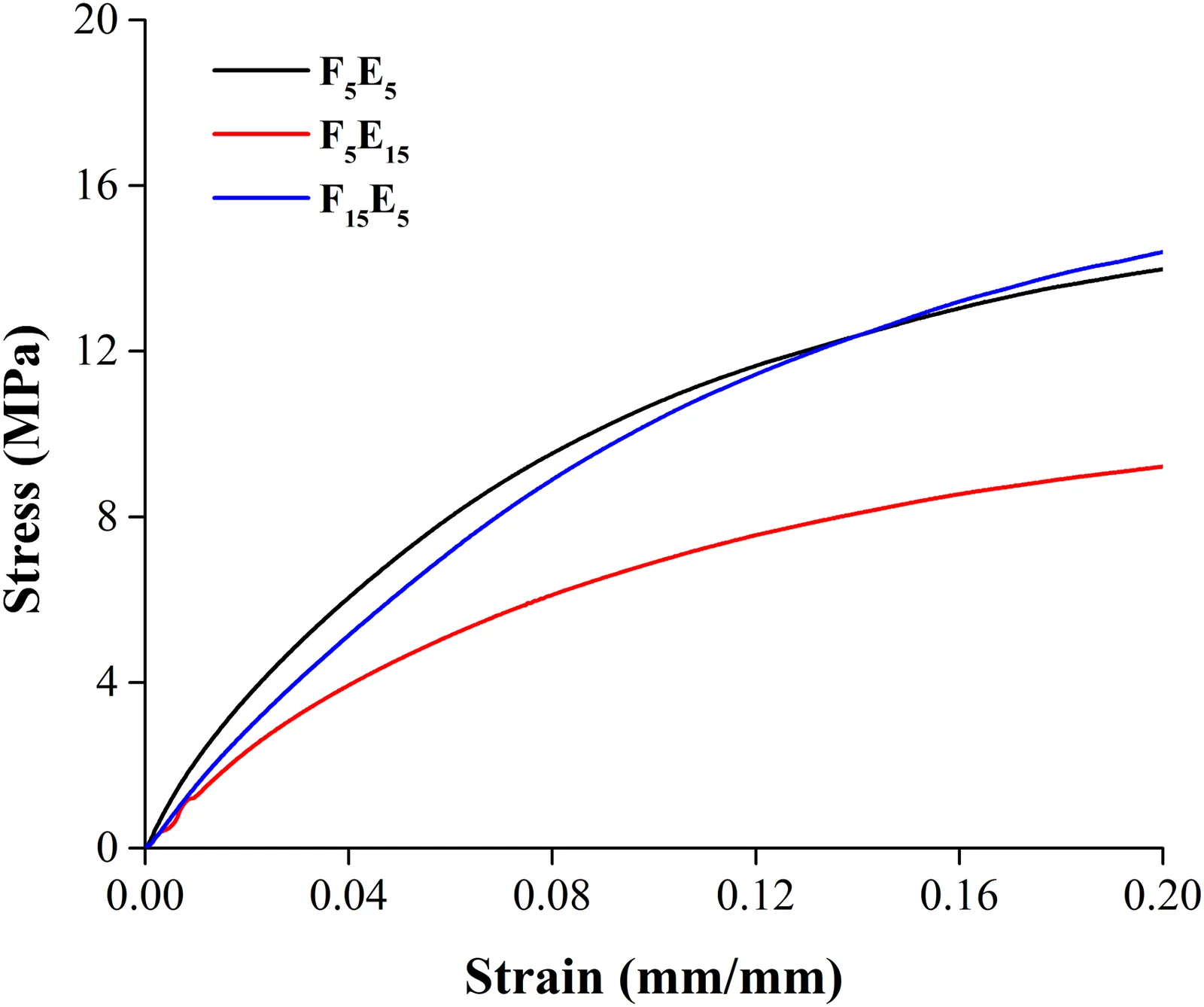
Stress-strain curves of PVA-Cassava fibre composite filaments.

Although the FTIR spectra showed similar chemical profiles among the composite filaments, clear differences in mechanical behaviour were observed among the formulations (Table 3 and Fig. 4). The reference formulation, F_5_E_5_, exhibited the highest apparent Young's modulus ($E_{\mathrm{app}}$; 164.77 ± 19.67 MPa), indicating the greatest stiffness among the tested formulations. In contrast, F_5_E_15_, which had a higher Eudragit® NE 30 D proportion and a corresponding lower PVA proportion, showed significantly lower maximum force ($F_{\max}$), tensile strength (TS), and $E_{\mathrm{app}}$ values than F_5_E_5_ (*p* < 0.05). The stress-strain curve of F_5_E_15_ also displayed a lower initial slope, indicating that the filament underwent greater deformation under tensile loading. This behaviour may be attributed to the higher Eudragit® NE 30 D proportion and the corresponding reduction in PVA content, together with the potential contribution of plasticising components present in the commercial Eudragit® NE 30 D dispersion. This observation is consistent with previous studies showing that increasing Eudragit® NE 30 D content significantly decreased tensile strength and Young's modulus due to the inherent flexibility of this neutral acrylate copolymer at room temperature (El-Malah and Nazzal, 2008).

**Table 3:** Mechanical properties and theophylline content of PVA-Cassava fibre composite filaments.

Formulation	$F_{\max}$ (N ± SD)	TS (MPa ± SD)	$E_{\mathrm{app}}$ (MPa ± SD)	Theophylline content (% ± SD)
F_5_E_5_	29.29 ± 2.68 ^a^	15.37 ± 0.78 ^a^	164.77 ± 19.67 ^a^	102.2 ± 1.1 ^a^
F_5_E_15_	17.92 ± 0.63 ^b^	9.79 ± 0.37 ^b^	114.56 ± 7.95 ^b^	99.3 ± 5.3 ^a^
F_15_E_5_	30.84 ± 0.71 ^a^	17.22 ± 0.32 ^c^	141.52 ± 6.37 ^c^	99.7 ± 7.3 ^a^

Note: Within the same column, values with different superscript letters are significantly different (*p* < 0.05), whereas values with the same superscript letter are not significantly different.

In comparison, F_15_E_5_, which had a higher Cassava fibre proportion and a corresponding lower PVA proportion than F_5_E_5_, demonstrated a distinct mechanical response. Its $E_{\mathrm{app}}$ value (141.52 ± 6.37 MPa) was significantly lower than that of F_5_E_5_ but remained significantly higher than that of F_5_E_15_ (*p* < 0.05), indicating an intermediate stiffness between the two formulations. Nevertheless, the F_15_E_5_ exhibited a comparable $F_{\max}$ value to F_5_E_5_ and significantly higher TS (*p* < 0.05). These experimental findings indicate that the higher Cassava fibre proportion reduced filament stiffness while maintaining tensile strength. No filament fracture was observed during tensile testing under the applied test conditions. Similar changes in mechanical behaviour at higher natural fibre loadings have been reported in fibre-reinforced polymer composites (Hargitai et al., 2008). This combination of reduced stiffness and maintained tensile performance may be beneficial for filament handling and subsequent FDM printing (Nasereddin et al., 2018).

Regarding theophylline content in the composite filaments, all formulations exhibited mean values close to the theoretical drug loading, ranging from 99.3% to 102.2% (Table 3). No significant difference was observed among the formulations (*p* > 0.05), indicating that increasing either Eudragit® NE 30 D or Cassava fibre content did not significantly affect the overall drug loading of the extruded filaments.

Although the mean drug content was comparable among all formulations, differences in the variability of drug content measurements were observed. Notably, F_5_E_5_ showed the lowest SD value, whereas F_5_E_15_ and F_15_E_5_ exhibited slightly higher variability. This trend may be associated with changes in the local distribution of formulation components following the incorporation of higher proportions of Eudragit® NE 30 D or Cassava fibre, although the composite filaments remained generally homogeneous overall. Nevertheless, the theophylline contents of all formulations remained close to the theoretical drug loading (∼100%), indicating that these local variations did not substantially affect overall drug incorporation. These findings further suggest that the pre-mixing procedure and melt mixing within the single-screw HME were sufficient to maintain the intended drug loading across all formulations (Maniruzzaman et al., 2012; Repka et al., 2013).

### 3.2 FDM printability and structural characteristics of the 4D-printed constructs

#### 3.2.1 Printability, structural fidelity, and theophylline content uniformity

**Fig. 5:**
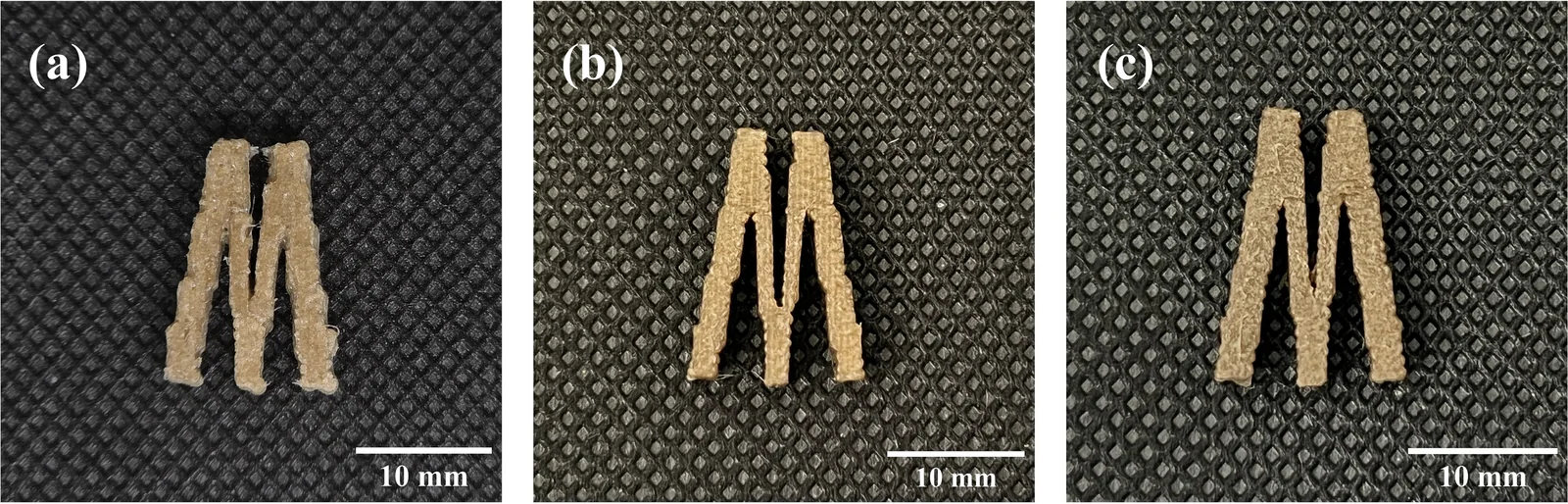
FDM-printed M-shaped constructs of (a) F_5_E_5_, (b) F_5_E_15_ and (c) F_15_E_5_ formulations.

All composite filaments were successfully processed *via* FDM to fabricate M-shaped constructs with the intended overall geometry (Fig. 5). The measured width, length, and thickness of all 4D-printed constructs closely matched the corresponding CAD dimensions (Table 4), demonstrating good dimensional accuracy. However, minor irregularities were observed along the printed contours, indicating some variation in structural fidelity. In addition, no major printing defects, including incomplete material deposition, structural collapse, or visible warping, were observed under the selected printing conditions. Notably, although F_15_E_5_ exhibited a rougher filament surface prior to printing, as described in Section 3.1.1, the resulting 4D-printed constructs showed dimensional accuracy and shape fidelity comparable to those of F_5_E_5_ and F_5_E_15_. These findings indicate that the compositional modifications did not adversely affect FDM processing or the reproducible fabrication of the designed M-shaped constructs.

**Table 4:** Dimensional accuracy and theophylline content of 4D-printed constructs.

Formulation	Shape fidelity after printing in each dimension (% ± SD)			Theophylline content (% ± SD)
	Width	Length	Thickness	
F_5_E_5_	101.0 ± 1.0	100.6 ± 0.3	99.4 ± 0.8	102.7 ± 8.2
F_5_E_15_	100.3 ± 0.9	100.5 ± 0.4	99.1 ± 1.0	100.4 ± 6.3
F_15_E_5_	100.0 ± 0.5	100.6 ± 0.4	99.3 ± 0.4	102.2 ± 3.1

Note: Each dimension of the M-shaped constructs was compared against the CAD model width as 12.64 mm, length as 18.35 mm, and height as 4.00 mm.

Additionally, the theophylline contents of the 4D-printed constructs remained close to the theoretical drug loading, ranging from 100.4% to 102.7% (Table 4). Importantly, all 4D-printed constructs contained theophylline within 90.0–110.0% of the theoretical contents, which falls within the acceptance range described by the United States Pharmacopeia (United States Pharmacopeial Convention (USPC), 2024). Furthermore, the drug content values were also comparable to those of the corresponding extruded filaments, suggesting that the additional thermal exposure during FDM printing did not result in significant drug loss. These findings verify the excellent thermal stability of theophylline throughout the consecutive HME-FDM fabrication processes.

#### 3.2.2 Solid-state and thermal characteristics of the 4D-printed constructs

To elucidate the physical state of the active ingredient and the crystalline nature of the polymeric matrix following consecutive thermal HME-FDM processing, X-ray diffraction (XRD) and differential scanning calorimetry (DSC) analyses were performed.

**Fig. 6:**
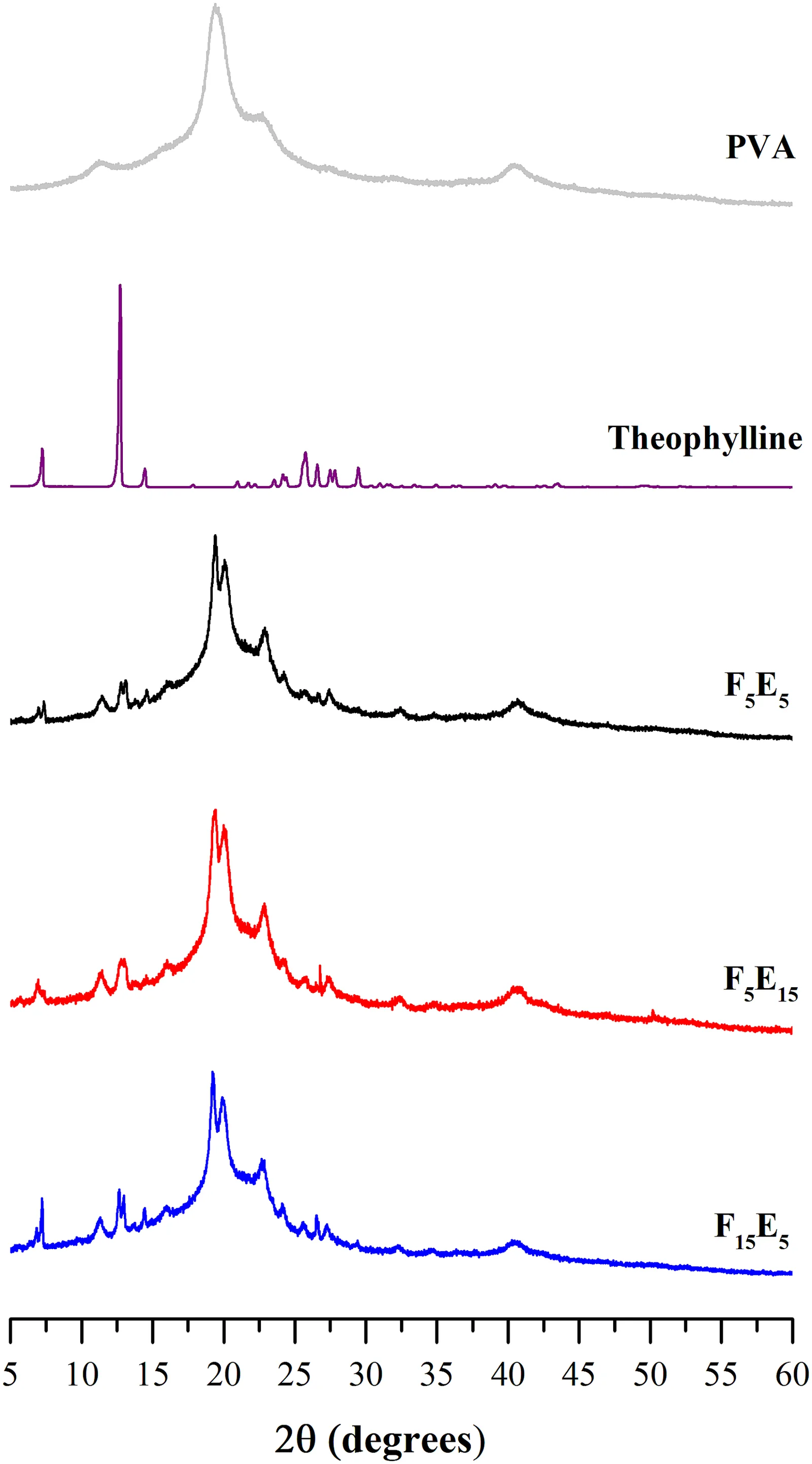
XRD patterns of pure PVA, raw theophylline, and 4D-printed constructs.

As shown in Fig. 6, raw theophylline exhibited a highly crystalline diffraction pattern with characteristic reflections at approximately 2θ = 7.2°, 12.7°, and 14.4°, corresponding to its anhydrous crystalline form (Airaksinen et al., 2004; Sandler et al., 2010). In contrast, pure PVA powder displayed a typical semi-crystalline pattern, characterised by a dominant reflection centred at approximately 2θ = 19.4°, a secondary shoulder near 22.7°, as well as a broad diffraction peak around 40.4° (Bozdoğan et al., 2020; Kovtun et al., 2024; Ricciardi et al., 2004).

Following HME-FDM processing, all 4D-printed constructs maintained the characteristic PVA-associated diffraction peak at approximately 19.4°, indicating that the semi-crystalline nature of the PVA phase was preserved after processing. In addition, weak diffraction peaks associated with crystalline theophylline were also detected in all formulations, although with substantially lower intensity compared with the raw drug. This reduction in peak density may be attributed to the low drug loading, dilution within the polymer matrix, and possible disruption of the original crystal structure during thermal processing. Moreover, because the selected HME-FDM processing temperature was below the melting temperature of theophylline (271 °C), complete melting of the drug was unlikely to occur, which may have contributed to the retention of residual crystallinity after processing. These findings suggest that theophylline remained partially crystalline within the 4D-printed constructs rather than undergoing complete amorphisation, consistent with previous reports describing residual crystallinity of theophylline after thermal processing in polymeric matrices (Lee et al., 2026; Tan et al., 2019).

**Fig. 7:**
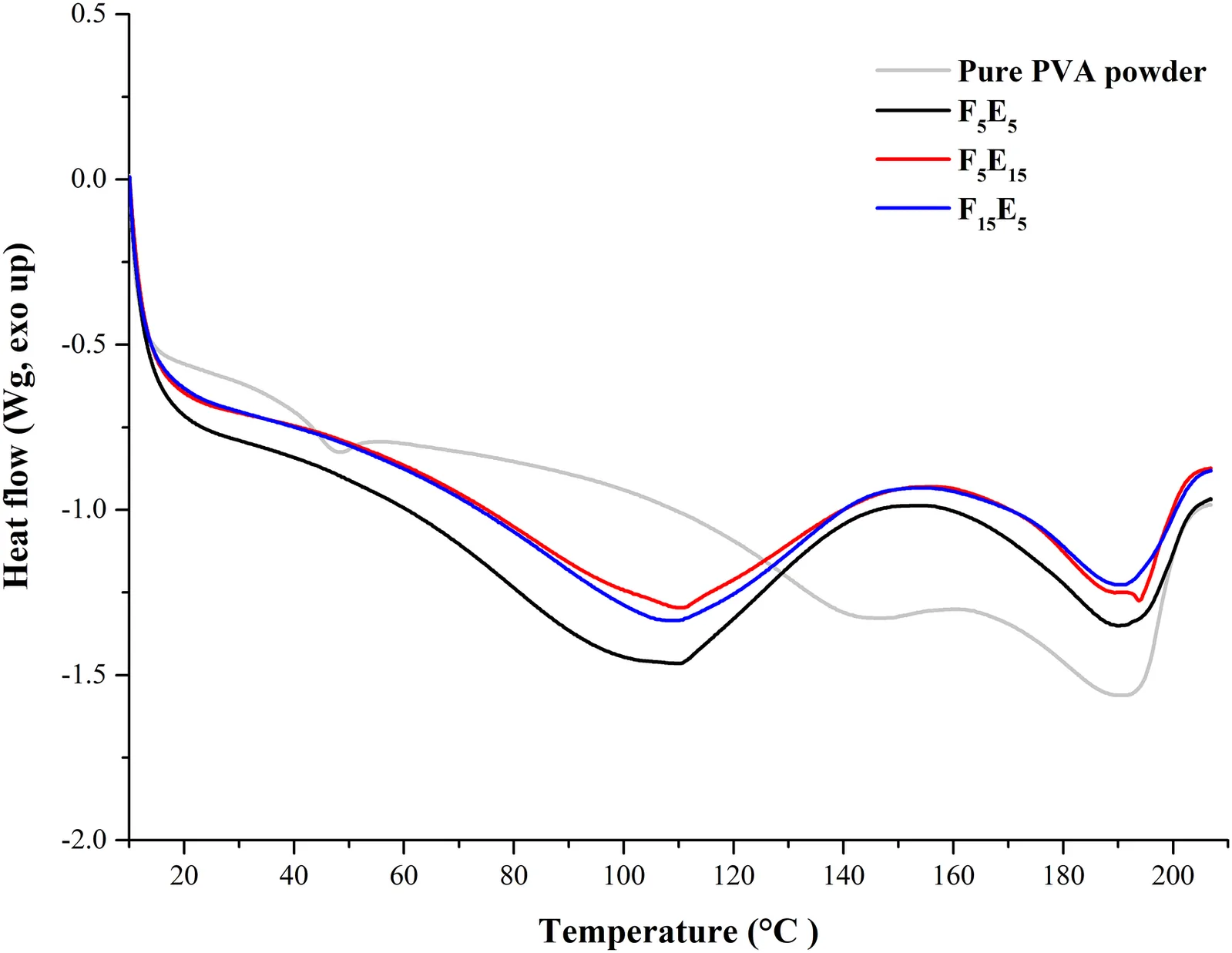
DSC thermograms of pure PVA powder and 4D-printed constructs.

DSC thermograms (Fig. 7) further corroborated the XRD findings, as all 4D-printed constructs exhibited distinct PVA melting endotherms, confirming the presence of crystalline PVA domains after HME–FDM processing. Pure PVA showed a melting endotherm at 192.53 °C, whereas F_5_E_5_, F_5_E_15_, and F_15_E_5_ exhibited melting peaks at 190.20, 193.93, and 191.08 °C, respectively. These melting temperatures are consistent with the reported melting transition of partially hydrolysed PVA at approximately 190 °C (Mallakpour and Rashidimoghadam, 2017). The selected processing temperature of 200 °C was therefore appropriate for enabling sufficient softening and processing of the PVA-based composite during HME-FDM fabrication.

To further compare the melting behaviour across formulations with different PVA contents, melting enthalpies ($∆H_{m}$) were normalised to the dry-basis PVA fraction of each construct. The normalised $∆H_{m}$ values were found to be 34.65, 33.58, and 33.05 J/g for F_5_E_5_, F_5_E_15_, and F_15_E_5_, respectively. The comparable melting peak positions and PVA-normalised melting enthalpies observed in this study indicate that increasing Eudragit® NE 30 D or Cassava fibre content did not substantially alter the thermal behaviour of the PVA phase after HME-FDM processing. This preservation of PVA melting behaviour provides a consistent thermal basis for subsequent shape-programming evaluation.

Besides the melting transition of the PVA phase, all 4D-printed constructs exhibited a broad low-temperature endothermic region between approximately 60 and 130 °C. This thermal event may be associated with moisture-related processes arising from the hygroscopic nature of PVA and Cassava fibre components, together with possible contributions from other formulation components. As the samples were analysed without additional drying prior to DSC measurement, residual moisture may have contributed to this broad endothermic region and obscured weaker thermal transitions. Consequently, a distinct glass transition temperature (T_g_) of PVA could not be clearly identified in the 4D-printed constructs, although a transition at approximately 42.85 °C was observed in the raw PVA thermogram (Abazari et al., 2025).

### 3.3 Shape programming, *in vitro* transformation and swelling behaviour

#### 3.3.1 Shape programming and capsule loading

For shape programming, the 4D-printed constructs were heated to sufficient polymer mobility for compression into a temporary compact shape (Spiegel et al., 2022). Although a distinct T_g_ could not be clearly identified in the 4D-printed constructs, the thermal behaviour of raw PVA was used as a practical reference for selecting the programming temperature range. During preliminary trials, programming temperatures above the Tg of raw PVA and different holding times were evaluated to identify conditions that allowed sufficient deformation and temporary shape fixation. At lower temperatures or shorter holding times, the constructs could not be completely compressed or retain the temporary shape after cooling. Based on these observations, 90 °C for 10 min under a 100-g load was selected for subsequent shape programming experiments.

Under the selected programming condition, the results demonstrated that F_5_E_5_ fractured during compression and could not be fixed into the temporary compact shape. This behaviour may be associated with its comparatively high stiffness, as F_5_E_5_ exhibited the highest $E_{\mathrm{app}}$ value among the tested formulations (Table 3). The relatively rigid matrix may have limited its ability to accommodate the localised deformation imposed during compression of the M-shaped construct, thereby resulting in brittle fracture rather than stable plastic deformation during shape programming. Compared with the relatively gradual deformation experienced by the filament during FDM feeding, compression of the 4D-printed construct imposed more complex bending and compressive stresses at the folded regions. In addition, the layer-by-layer structure of FDM-printed constructs may introduce interlayer regions with lower mechanical strength, which could contribute to fracture during compression (Duty et al., 2018; Thumsorn et al., 2022).

**Fig. 8:**
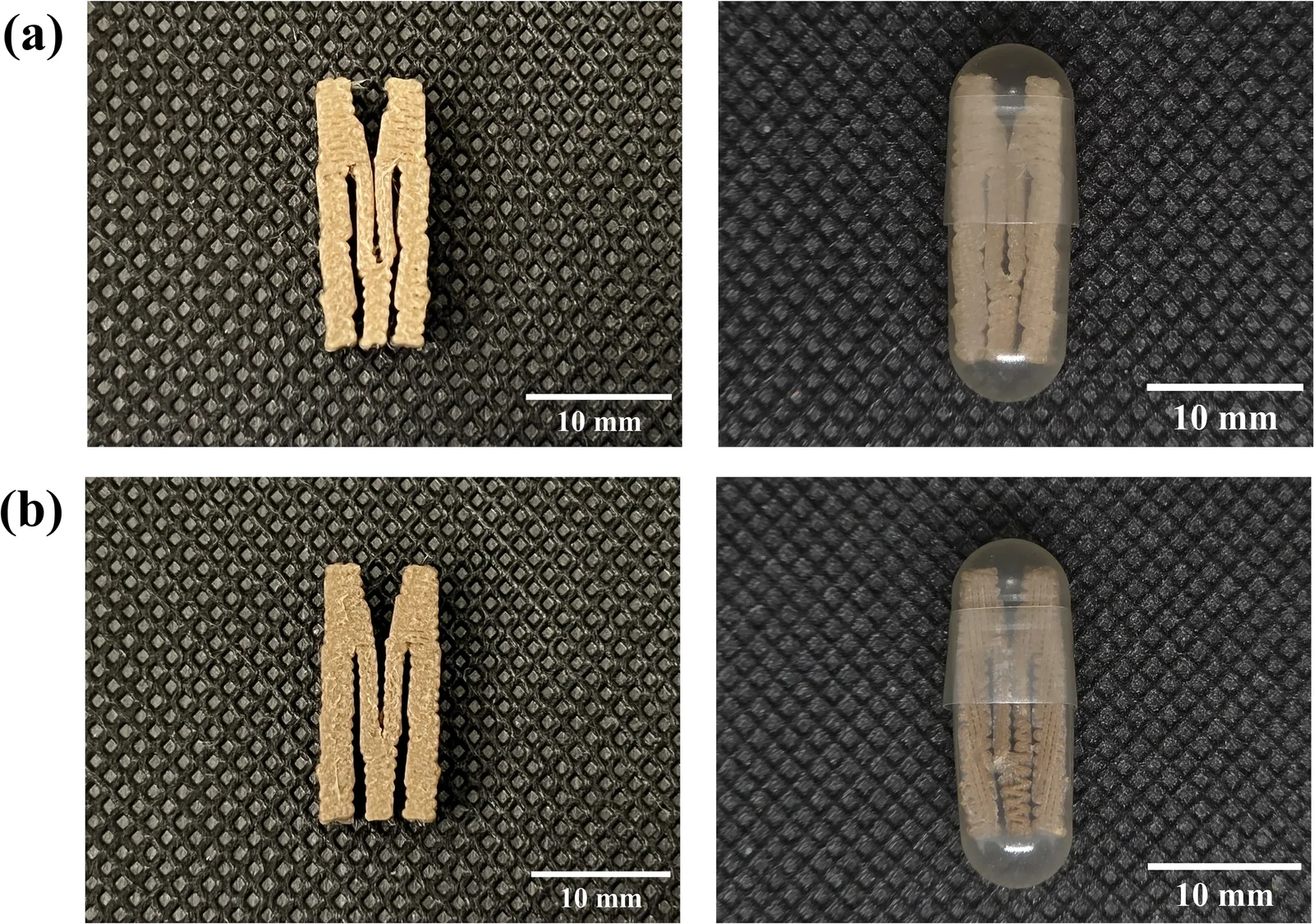
Representative photographs of (a) F_5_E_15_ and (b) F_15_E_5_ 4D-printed constructs after fixation in the temporary compact shape (left) and after loading into capsules (right).

In contrast, increasing either Eudragit® NE 30 D or Cassava fibre content to 15% *w*/w improved the flexibility of the composite constructs, enabling successful shape programming and capsule loading without visible structural damage (Fig. 8). For F_5_E_15_, the higher Eudragit® NE 30 D content likely increased the ductility of the PVA-based matrix due to the flexible characteristics of Eudragit® NE 30 D, allowing the construct to undergo greater deformation during compression (Suksaeree et al., 2021). For F_15_E_5_, the increased Cassava fibre content may have enhanced the mechanical integrity of the composite matrix by reinforcing the polymer network and redistributing localised stresses during compression, thereby reducing the likelihood of fracture (Singleton et al., 2003). Therefore, both formulations provided improved resistance to compression-induced failure through different mechanisms and were selected for subsequent *in vitro* shape recovery and release studies.

#### 3.3.2 *In vitro* shape transformation and swelling behaviour

Upon immersion in simulated gastric conditions, both capsule-loaded 4D-printed constructs (F_5_E_15_ and F_15_E_5_) exhibited transient floating behaviour during the initial 10 min (Fig. S1). At approximately 15 min, the HPMC capsules had completely disintegrated, after which the constructs sank into the dissolution medium. This initial floating behaviour may be attributed to air retained within the capsule shells and/or the compact geometry of the 4D-printed constructs. Following capsule disintegration, medium penetration into the constructs and the release of trapped air may have resulted in loss of buoyancy and subsequent sinking. This transient buoyancy may provide an initial advantage in terms of gastroretention during deployment, as the 4D-printed constructs were still undergoing shape recovery and had not yet reached their expanded gastroretentive configuration.

Shape recovery was also monitored from the initial immersion time points. The measurable recovery ratios at 5 and 10 min indicate that both formulations had already begun to expand gradually while the capsule shells were still undergoing disintegration. As shown in Table 5, both formulations exhibited progressive shape recovery under simulated gastric conditions. F_15_E_5_ showed significantly higher recovery ratios than F_5_E_15_ at 5, 10, and 15 min. At 30 min, F_15_E_5_ exhibited significantly greater recovery, reaching 93.3 ± 3.6%, compared with 78.2 ± 5.3% for F_5_E_15_. The observed transformation of both formulations can be attributed to the hydration-induced shape-memory behaviour of the semi-crystalline PVA-based matrix. Upon immersion, water penetrated the hydrophilic PVA matrix, plasticising the polymer chains and increasing their mobility. This allowed the programmed amorphous regions to relax towards the original M-shaped configuration, while the crystalline domains acted as physical anchoring points that preserved the permanent shape memory and provided the necessary restoring force (Chen et al., 2016; Wang et al., 2022). The faster recovery observed for F_15_E_5_ may be associated with the higher Cassava fibre content. The higher fibre content may have introduced additional fibre-matrix interfaces within the composite structure, which could facilitate water penetration into the matrix and accelerate hydration-induced polymer relaxation during immersion (Muñoz and García-Manrique, 2015).

**Table 5:** Shape recovery ratios of capsule-loaded 4D-printed constructs.

Formulation	Shape recovery ratios at respective time points (% ± SD)				
	5 min	10 min	15 min	20 min	30 min
F_5_E_15_	1.5 ± 0.3 ^a^	8.1 ± 0.4 ^a^	29.7 ± 2.1 ^a^	61.3 ± 6.6 ^a^	78.2 ± 5.3 ^a^
F_15_E_5_	4.5 ± 0.2 ^b^	11.9 ± 0.3 ^b^	41.6 ± 2.5 ^b^	60.3 ± 1.2 ^a^	93.3 ± 3.6 ^b^

Note: Within the same column, values with different superscript letters are significantly different (*p* < 0.05), whereas values with the same superscript letter are not significantly different.

To further examine the relationship between hydration and shape recovery, apparent swelling was evaluated in parallel (Table 6). F_5_E_15_ exhibited a continuous increase in apparent swelling throughout the observation period and showed significantly higher swelling ratios than F_15_E_5_ at all time points. The greater swelling of F_5_E_15_ may be attributed to its higher Eudragit® NE 30 D content, as this water-insoluble neutral acrylate copolymer can contribute to a flexible polymer network capable of retaining absorbed water within the construct (Bölcskei et al., 2012). However, the greater swelling of F_5_E_15_ did not result in enhanced shape recovery. This may be because excessive hydration-induced expansion of the compacted construct increased resistance to unfolding, thereby limiting the recovery towards the original M-shaped geometry.

**Table 6:** Apparent swelling ratios of 4D-printed constructs.

Formulation	Apparent swelling ratios at respective time points (% ± SD)				
	5 min	10 min	15 min	20 min	30 min
F_5_E_15_	17.8 ± 9.0 ^a^	21.5 ± 6.2 ^a^	27.0 ± 8.2 ^a^	37.0 ± 5.8 ^a^	40.7 ± 8.7 ^a^
F_15_E_5_	3.9 ± 1.7 ^b^	21.0 ± 9.5 ^a^	15.3 ± 3.2 ^b^	8.0 ± 1.1 ^b^	6.0 ± 2.2 ^b^

Note: Within the same column, values with different superscript letters are significantly different (*p* < 0.05), whereas values with the same superscript letter are not significantly different.

In contrast, F_15_E_5_ showed a rapid increase in swelling during the initial stage, followed by a gradual decrease after 10 min. This behaviour may be associated with the higher Cassava fibre content, which increased water accessibility within the composite structure and promoted hydration of the PVA matrix. As the PVA matrix hydrated, the presence of fibre-matrix interfaces may have facilitated water penetration into the construct, promoting progressive erosion around the outer regions of the M-shaped structure and gradual exposure or shedding of the embedded fibres (Markl and Zeitler, 2017). Based on visual observation, the constructs began to show progressive deformation after 30 min, although the overall expanded M-shaped configuration remained recognisable for approximately 2 h. Beyond this period, continued deformation and erosion resulted in gradual loss of the original geometry. From a gastroretentive perspective, this short-term maintenance of the expanded configuration supports the potential of the present design to prolong gastric residence, although further optimisation is required to improve structural persistence and confirm the actual gastroretentive performance *in vivo*.

### 3.4 *In vitro* theophylline release and release kinetic analysis

**Fig. 9:**
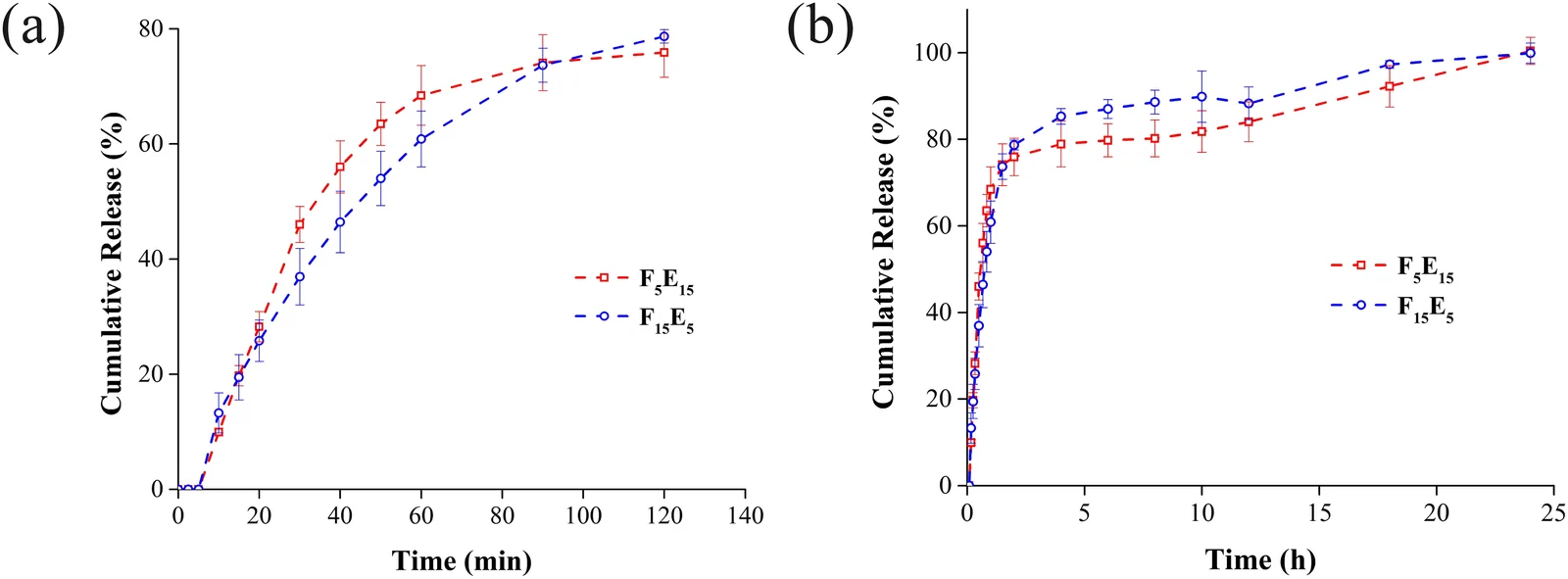
*In vitro* theophylline release profiles from capsule-loaded 4D-printed constructs (a) during first 2 h and (b) over 24 h.

The *in vitro* theophylline release profiles from the capsule-loaded 4D-printed constructs are presented in Fig. 9. Both formulations exhibited an initial lag phase of approximately 5 min, during which negligible drug release was detected. This delay is directly related to the time required for the HPMC capsule to undergo initial disintegration under simulated gastric conditions. Following this lag phase, a rapid increase in cumulative drug release was observed for both constructs. During the first hour, F_5_E_15_ showed a slightly faster release rate compared to F_15_E_5_. This behaviour may be attributed to its lower fibre content, which reduced the tortuosity of the diffusion pathways and allowed more rapid diffusion of theophylline through the hydrated polymer matrix. However, the release profiles crossed over at approximately 1.5 h, after which F_15_E_5_ exhibited greater cumulative drug release than F_5_E_15_ throughout the remainder of the study. This trend is consistent with the swelling study (Section 3.3.2), where F_15_E_5_ showed progressive matrix erosion and fibre shedding, gradually exposing more entrapped theophylline to the dissolution medium.

Overall, both formulations showed a similar biphasic release pattern, characterised by a pronounced initial release followed by a considerably slower release phase. Approximately 70–80% of theophylline was released within the first 2 h, with the remaining drug released more gradually thereafter and release approaching completion at 24 h. This release pattern indicates that the current formulations did not provide a gradual and sustained release throughout the entire study period. However, drug release was not completed within the initial 2 h and continued progressively thereafter, representing a more extended-release profile than previously reported for PVA-based expandable systems, in which complete drug release occurred within 2 h (Melocchi et al., 2019).

To further investigate the release mechanism, the dissolution data were analysed using the Korsmeyer-Peppas model, which provided a good fit for both formulations, with R^2^ values greater than 0.98 (Table 7). The release exponent (n) was 0.86 for F_5_E_15_ and 0.81 for F_15_E_5_, indicating anomalous (non-Fickian) transport. These findings suggest that theophylline release involved a combination of diffusion through the hydrated matrix and polymer relaxation and/or erosion. This interpretation is consistent with the swelling and shape-transformation observations in Section 3.3.2 (Table 5). The higher Eudragit® NE 30 D content of F_5_E_15_ was associated with greater swelling and water retention within the matrix, whereas the higher Cassava fibre content of F_15_E_5_ was associated with progressive matrix erosion and fibre shedding during immersion. These formulation-dependent structural changes may therefore contribute to the observed non-Fickian release behaviour.

**Table 7:** Release kinetic data of the 4D-printed constructs.

Release Kinetic Model	Kinetic Parameter	Formulation	
		F_5_E_15_	F_15_E_5_
	$R^{2}$	0.9823	0.9978
Korsmeyer-Peppas	$k_{p}$	2.20	2.30
	n	0.86	0.81

Nevertheless, further formulation optimisation is required to reduce the pronounced initial release and achieve a more gradual release profile that is better aligned with prolonged gastroretentive drug delivery. Future studies could explore blending PVA with more hydrophobic shape-memory polymers, such as PLA or PCL, to improve matrix persistence and modulate drug release while maintaining shape-memory behaviour under physiologically relevant conditions.

## 4 Conclusion

This study demonstrated the feasibility of using sustainable PVA-Cassava fibre composite filaments as programmable materials for FDM-based 4D printing of expandable gastroretentive drug delivery systems. All formulations were successfully extruded into continuous filaments and printed into M-shaped constructs with high dimensional accuracy and accurate theophylline loading. However, the reference formulation (F_5_E_5_) fractured during thermomechanical programming, indicating that its relatively rigid matrix was unsuitable for conversion into a capsule-compatible temporary configuration. In contrast, increasing either Eudragit® NE 30 D or Cassava fibre content to 15% *w*/w improved construct flexibility and enabled successful shape programming and capsule loading without visible structural failure. In this study, the programmed constructs provided a sequential gastroretentive strategy, in which capsule-mediated transient buoyancy supported the early deployment stage, followed by water-triggered recovery towards the expanded geometry after capsule disintegration. Among the programmable formulations, F_15_E_5_ exhibited the most rapid and complete shape recovery, achieving 93.3 ± 3.6% recovery within 30 min, whereas F_5_E_15_ showed greater swelling and matrix persistence. These compositional differences further influenced theophylline release behaviour, with the high-fibre formulation (F_15_E_5_) promoting progressive erosion and fibre shedding, while the high Eudragit® formulation (F_5_E_15_) maintained a more persistent hydration- and swelling-controlled matrix. Overall, these findings highlight the potential of Cassava fibre-reinforced PVA composites as sustainable and tuneable platforms for 4D-printed gastroretentive drug delivery systems, enabling programmable shape transformation with composition-dependent drug release behaviour. Despite these promising findings, further optimisation should focus on improving structural persistence and achieving a more gradual release profile, together with assessing the storage stability and capsule integrity of the final product, evaluating the recovery force generated during unfolding, and confirming gastroretentive and therapeutic performance under biorelevant and *in vivo* conditions.

## CRediT authorship contribution statement

**Pattaraporn Panraksa:** Writing – review & editing, Writing – original draft, Validation, Project administration, Methodology, Investigation, Formal analysis, Conceptualization. **Krit Sengtakdaed:** Writing – original draft, Visualization, Methodology, Investigation, Formal analysis. **Ploynapat Pornngam:** Visualization, Investigation, Formal analysis. **Kittisak Jantanasakulwong:** Resources, Methodology. **Bin Zhang:** Writing – review & editing, Methodology, Investigation, Formal analysis. **Claire-Hélène Brachais:** Writing – review & editing, Methodology, Investigation, Formal analysis. **Odile Chambin:** Writing – review & editing, Writing – original draft, Supervision, Methodology, Investigation, Conceptualization. **Pensak Jantrawut:** Writing – review & editing, Writing – original draft, Supervision, Methodology, Investigation, Conceptualization.

## Funding sources

This research was supported by the Fundamental Fund 2026 10.13039/501100002842Chiang Mai University and the NSRF *via* the Research and Innovation Acceleration Agency for Competitiveness and Area Development (RCAD) (Program Management Unit for Frontier Brainpower and Future Industries) [Grant number B49G690124].

## Declaration of competing interest

The authors declare the following financial interests/personal relationships which may be considered as potential competing interests: Pattaraporn Panraksa reports financial support was provided by 10.13039/501100002842Chiang Mai University. If there are other authors, they declare that they have no known competing financial interests or personal relationships that could have appeared to influence the work reported in this paper.

## Data Availability

Data will be made available on request.
