# Supplementary material for: FDM-based 4D printing of sustainable PVA-Cassava fibre composites: a programmable platform for smart gastroretentive drug delivery
Source: Int J Pharm X. 2026 Sep 13;12:100665. doi: 10.1016/j.ijpx.2026.100665 (PMC13598595; doi:10.1016/j.ijpx.2026.100665)
Supplement: Supplementary file 1 [file mmc1.docx]

**Supplementary Materials: FDM-Based 4D Printing of Sustainable PVA-Cassava Fibre Composites: A Programmable Platform for Smart Gastroretentive Drug Delivery**

**Pattaraporn Panraksa ^a^, Krit Sengtakdaed ^a^, Ploynapat Pornngam ^a^, Kittisak Jantanasakulwong ^b,c^, Bin Zhang ^d^, Claire-Hélène Brachais ^e^, Odile Chambin ^f,g,*^, and Pensak Jantrawut ^a,c,*^**

**Fig. S1.** *In vitro* floating of capsule-loaded F_5_E_15_ (left) and F_15_E_5_ (right) 4D-printed constructs in simulated gastric condition (0.1 N HCl, 37 ± 0.5 °C).
